# The "lessons" of the Australian "heroin shortage"

**DOI:** 10.1186/1747-597X-1-11

**Published:** 2006-05-02

**Authors:** Louisa Degenhardt, Carolyn Day, Stuart Gilmour, Wayne Hall

**Affiliations:** 1 National Drug and Alcohol Research Centre, University of New South Wales, SYDNEY, NSW 2052, Australia; 2 National Centre in HIV Epidemiology and Clinical Research, University of New South Wales, SYDNEY, NSW 2052, Australia; 3 Office of Public Policy and Ethics, Institute for Molecular Bioscience, University of Queensland, St Lucia, Brisbane, 4072, Australia

## Abstract

Heroin use causes considerable harm to individual users including dependence, fatal and nonfatal overdose, mental health problems, and blood borne virus transmission. It also adversely affects the community through drug dealing, property crime and reduced public amenity. During the mid to late 1990s in Australia the prevalence of heroin use increased as reflected in steeply rising overdose deaths. In January 2001, there were reports of an unpredicted and unprecedented reduction in heroin supply with an abrupt onset in all Australian jurisdictions. The shortage was most marked in New South Wales, the State with the largest heroin market, which saw increases in price, dramatic decreases in purity at the street level, and reductions in the ease with which injecting drug users reported being able to obtain the drug. The abrupt onset of the shortage and a subsequent dramatic reduction in overdose deaths prompted national debate about the causes of the shortage and later international debate about the policy significance of what has come to be called the "Australian heroin shortage". In this paper we summarise insights from four years' research into the causes, consequences and policy implications of the "heroin shortage".

## Background

Heroin use causes considerable harm to individual users through the development of dependence upon the drug, fatal and nonfatal overdose, mental health problems, and blood borne virus transmission. It also adversely affects the community through drug dealing, property crime and reduced public amenity. Recent decades have seen an increase in the prevalence of heroin use in many developed (and increasingly, developing) countries as reflected in rising overdose deaths [1].

Australia had a particularly steep increase in heroin overdose deaths between the mid and late 1990s with the result that in 1999 there were 1116 opioid overdose deaths among those aged 15 to 54 years [2]. Such deaths accounted for one in eight deaths among young Australians aged 15–24 years at that time [2]. There were also substantial rises in the number of people: treated for heroin dependence, arrested for heroin offences, and diagnosed with hepatitis C infections [3-5]. It had been estimated that injection drug-related hepatitis C will become the largest cause of liver transplants in Australia [5].

**Figure 1:**
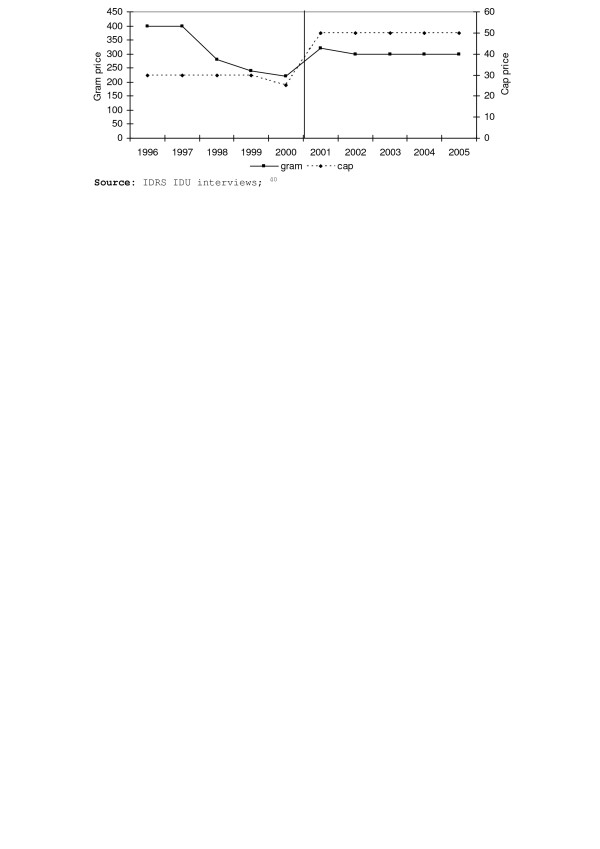
Median price (AUD) of a gram and “cap”(a street deal) of heroin estimated from IDU purchases, 1996 – 2005

**Figure 2:**
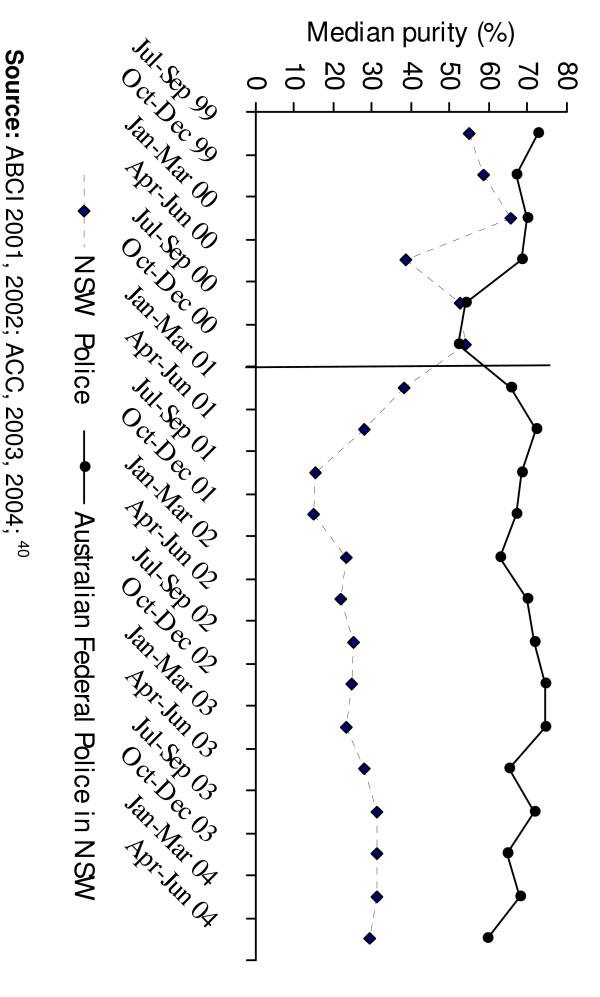
Purity of heroin seizures analysed in NSW, by quarter, 1999 – 2004

**Figure 3:**
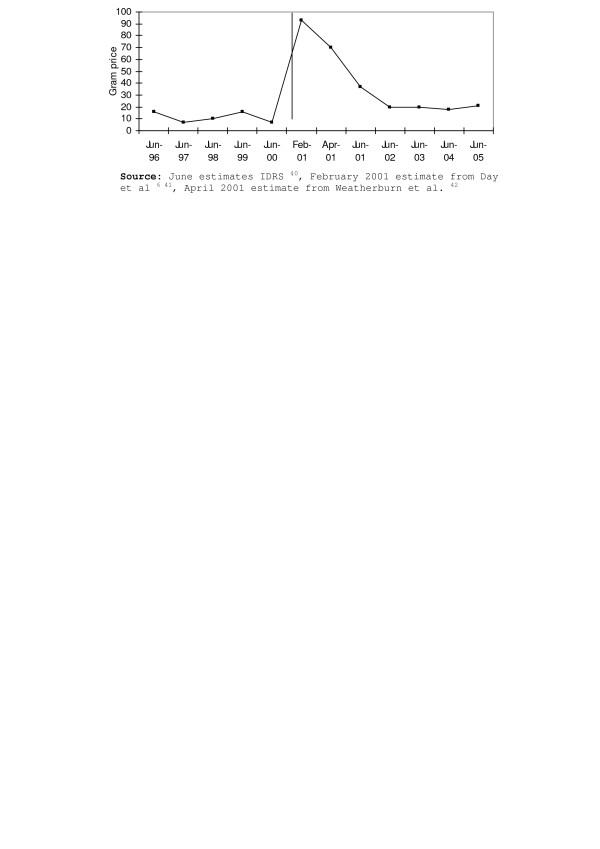
Proportion of IDU reporting that heroin had recently become more difficult to obtain, 1996-2005

In January 2001, the increase in overdose deaths was reversed by an unpredicted and unprecedented reduction in heroin supply that abruptly affected all Australian jurisdictions. The shortage was most marked in New South Wales, the State with the largest heroin market [6,7], where there were increases in price, dramatic decreases in purity at the street level, and reductions in the ease with which injecting drug users reported being able to obtain the drug (see Figures 1–3). The abrupt onset of the shortage and the dramatic reduction in overdose deaths prompted first national, then international debate, about the causes and the policy significance of what came to be called the "Australian heroin shortage" (e.g. [8-12]). In this paper we reflect on insights provided by four years' research into the causes, consequences and policy implications of the reduction in heroin supply.

## The impact of the heroin shortage on heroin use and heroin-related harm

**Figure 4:**
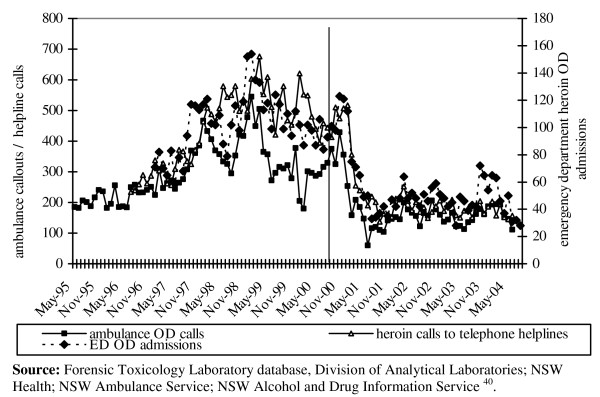
Number of heroin related ambulance callouts, heroin related emergency department admissions, and calls to telephone helplines about heroin, 1995-2004

The onset of the heroin shortage was followed by substantial reductions in multiple indicators of heroin use in the larger Australian heroin markets in NSW and Victoria (see Figure 4). The most notable and the most important effect was a 67% reduction in fatal and nonfatal opioid overdoses [13]. Deaths in Australia due to opioids declined from 1116 in 1999 to 386 in 2001 among those aged 15–54 years, and they have remained at this level in the three years since [13,14]. Hepatitis C notifications also decreased [15], whereas mathematical models of the epidemic [5] made in the preceding year had predicted an increase. This was probably due to a reduction in the extent of injecting drug use in major drug markets [16]. Street drug markets also reduced in size and drug sales became much less overt [17,18]. Changes in rates of property crime were not marked in Victoria and other states [7], but in NSW there was a short-lived spike in property crimes involving violence (perhaps related to increased cocaine use among IDU [19]), and a continuing fall (which had begun prior to the onset of the shortage) that has persisted since that time [18].

Regular drug injectors reported less frequent heroin use and more frequent injection of other drugs, such as cocaine in NSW, and amphetamines and benzodiazepines in Victoria [7,20,21]. Some of these IDU engaged in riskier forms of injecting and reported more drug-related harms [17,22,23] but there were no increases in deaths related to this other drug use [17,23-26]. We can be less confident about other harms caused by injecting drug use because the health consequences of psychostimulant drugs are not well captured in existing data collection systems. There were, however, significant impacts upon health services and local law enforcement, who began dealing with users exhibiting the behavioural effects of heavy psychostimulant use, and who increasingly reported significant polydrug use problems [17,22,27,28].

## Was the heroin shortage really a "shortage"?

The 1990s was a period of strong growth in heroin markets in Australia, with increases in the availability of the drug, the creation and expansion of street drug markets, and substantial rises in heroin related harms [29]. Dietze and Fitzgerald have argued that this period reflected a heroin "glut", the like of which had never before been seen in Australia [30]. They argued that the term "drought" implied a "normal" level of supply that was not in fact "normal" – the levels of supply were higher than ever before seen in Australia. They also argued that the improved monitoring of illicit drug markets from the mid 1990s may have increased perceptions of increased availability and then heightened awareness of the reduction in 2001. Finally, they argued that it was premature to draw conclusions about the reasons for the change in the market before establishing whether it was merely a return to pre-"glut" conditions [30]. Their paper has stimulated discussion in many quarters, and continued interest in the ideas presented therein warrants consideration in the light of the evidence we now have before us.

First, it is important to note that Dietze and Fitzgerald at no time disputed the fact that there was a large reduction in heroin supply at the beginning of 2001. They suggested that the change may have been exaggerated because it was well documented, and that it was important not to take the pre-shortage supply levels (those of the late 1990s) as "normal". In exploring the effects of a reduction in heroin supply the absolute levels pre and post the change are less important than the fact that there was a substantial reduction in supply with an abrupt onset. Availability and purity decreased and price increased [6,31] within a month in all Australian states, and this change was associated with statistically demonstrable reductions in heroin related harms (see below) [32,33].

Second, the heroin "glut" in the mid 1990s provided important information about the heroin market that in turn provided potential explanations of why and how the shortage may have occurred. Specifically, it is likely that a relatively small number of high level trafficking groups in South East Asia targeted Australia as a destination country for heroin in the mid 1990s and used sophisticated and large-scale shipment methods to import unprecedented amounts of heroin. This was probably an important reason for the *increase *in heroin supply during this period [24,34]. Law enforcement success in detecting the methods of importation used by these groups (and the consequent operational successes in making large seizures in 1999–2000) probably contributed to decreasing heroin supply in 2001 by encouraging these groups to send heroin to other countries [34].

Third, the relative contribution of the pre-shortage "glut" and the shortage itself to heroin related harm can be investigated statistically. We conducted a principal component analysis (PCA) of 17 key indicator data series from NSW in order to isolate a small number of *uncorrelated *principal components that explain the majority of the variance over time in these indicators [32]. PCA is a useful tool for assessing the relative importance of different changes over time, since it sorts the underlying drivers of variance in the indicators (the principal components) according to the magnitude of their effect. The PCA 'loadings' are used to compare variables, with variables with positive loadings contrasted against variables with negative loadings. We conducted PCA with the months of the data sets treated as variables. The first principal component, which explained 47% of the variance across data series over time, clearly contrasted the months before January 2001 with the months after that time. The second principal component (which explained 8% of the variance) contrasted those months in the peak of the 'glut' (1999–2000) with those before and after. These components suggest that: a) the heroin shortage explained more variance in the data series than the 'glut', and b) the shortage and the glut were independent events (since the principal components were uncorrelated).

## Why did the shortage happen?

The explanations of this change in heroin supply have been debated by researchers and in the community at large [9-12,35]. One suggestion was that the "shortage" simply reflected a return to the level of heroin supply that prevailed before a heroin "glut" in the 1990s. As noted above, there is some support for the hypothesis that the heroin shortage was preceded by a huge growth in the size of heroin markets in Australia in the 1990s [3], but as noted above, this growth was statistically uncorrelated with the shortage of 2001.

We evaluated all hypotheses proposed to explain the shortage, and ruled out those that were implausible using data from dozens of interviews with State, national and international informants, as well as detailed data on the Australian and international heroin and other drug markets collected from published reports, law enforcement briefings and routine data collections [34].

We concluded that the shortage was probably due to a confluence of factors reflecting the complexity of the heroin market [34]. One of these factors was probably the increased success of high-level Australian drug law enforcement operations conducted nationally and internationally by the Australian Federal Police and Customs (in cooperation with other agencies internationally). These operations removed key individuals directing a small number of highly centralised drug trafficking networks that had supplied large amounts of heroin to Australia, and seized over 1000 kg of heroin in 2000 [34]. Changes in source countries (such as reduced heroin production or increased methamphetamine production) probably also played a role but these did not explain the abrupt onset or the sustained reduction in heroin supply that occurred in Australia at least a year before any shortages were reported in other countries that sourced heroin from these same regions.

## Was this a victory for supply reduction?

Some commentators have argued that the findings provide unequivocal support for superiority of supply reduction to other approaches to drug control such as harm reduction [see for example [36,37]]. This inference is mistaken because it ignores the fact that the reduction in heroin supply occurred in a setting in which harm reduction measures (such as increased treatment and needle and syringe programs) were well integrated with supply and demand reduction initiatives. Australia has an integrated illicit drug policy that includes harm and demand reduction measures [38] such as increasing treatment places for opioid dependence and widespread availability of needle and syringe programs. The documented benefits of the reduction in heroin supply in Australia therefore occurred against a background of harm and demand reduction initiatives that probably reduced the severity of some of the negative consequences of reduced heroin supply (such as drug substitution and higher risk injecting).

Furthermore, our conclusion was that *high-level *law enforcement operations that disrupted highly centralised drug importation networks were probably a contributory cause of the shortage [34]. This does not contradict other findings that law enforcement activities directed at the *lowest *levels of the drug market may have negative consequences for users [39,40].

Nor do our findings contradict other research evidence that r*outine *heroin seizures have little or no effect on heroin prices or heroin use [41,42]. The scale of seizures in Australia during 2000, for example, comprised 30% of estimated annual heroin consumption [43] compared with 10% in earlier studies of the effects of routine seizures on heroin price and availability. In addition, key persons in the limited number of centralised trafficking networks that controlled the market were arrested in these operations. These two factors probably combined to make Australia a less attractive destination for *large scale *heroin traffickers. The disruption of these centralised large scale drug distribution networks seems to have produced a return to the methods of importation that were used before the "glut", that is, multiple importations of smaller quantities of heroin using drug couriers and other methods. This has been reflected in the number of recently highly publicised arrests of Australian heroin "mules" in Australia, Indonesia, Hong Kong and Singapore.

## Implications for drug policy

The heroin shortage has demonstrated that it was possible, in the unique conditions that characterised heroin supply in Australia in the late 1990s, for drug law enforcement to play a role in substantially reducing supplies of a major drug of dependence. However, this outcome occurred in a unique context that may not be easily reproduced in most countries, specifically (i) a small number of highly centralised heroin importation networks, (ii) that were importing large quantities of heroin into Australia, (iii) an isolated island continent, (iv) that had a relatively small heroin market by world standards, and (v) in which IDU had good access to a wide range of treatment and harm reduction options.

The heroin shortage has also shown that supply reduction can result in drug market shock, increasing price and decreasing purity and availability. We are now aware that in such situations, dependent heroin users alter their drug consumption patterns – the shortage resulted in a clear reduction in heroin use and increase in the use of other drugs, albeit (in some instances) of a limited duration. This provides some evidence that demand for heroin is price-elastic, i.e. heroin consumption and expenditure is reduced when price increases [44]. The effects of the reduction in heroin use were difficult to disentangle from the effects of changes in the availability of other drugs, increased treatment uptake and retention, and drug substitution.

These market changes led to clear public health benefits including reduced overdose deaths and a possible reduction in injecting drug use and hepatitis C infections. It is the latter conclusions that have been considered by some the most contentious, but as we have argued previously, the benefits of the heroin shortage need to be interpreted in the context of existing harm and demand reduction initiatives which are ameliorated its impact on heroin users. Deaths attributable to opioid drug overdose have remained at the same level for three years post-shortage, but there has been a small increase in overdose deaths attributable to stimulant drug use [45].

These findings are consistent with what is known about the supply and control of licit drugs. There is good evidence to suggest that when the availability of legal drugs such as alcohol and tobacco are altered (through legal controls on availability and cost), that community level harms also alter as a result [46,47]. The literature on alcohol and tobacco also suggests that some groups are less affected by changes in availability than others [47], as was also suggested in the heroin shortage work [22,48].

The Australian heroin shortage provides good evidence that the integration of supply, demand and harm reduction measures can substantially reduce the harmful effects of injecting heroin use. It would have been very difficult to monitor these changes had we not had the benefit of established strategic early warning systems to document the changes we were able to examine.

## Authors' contributions

All authors 1) have made substantial contributions to conception; 2) have been involved in drafting the manuscript or revising it critically for important intellectual content; and 3) have given final approval of the version to be published.

## Competing interests

The author(s) declare that they have no competing interests.
